# Delayed Complex Spike Response Evoked by Conditioned Stimulus Encodes Movement Onset Time and Is Determined by Intrinsic Inferior Olive Properties

**DOI:** 10.3389/fnsys.2019.00050

**Published:** 2019-10-09

**Authors:** Yasmin Yarden-Rabinowitz, Yosef Yarom

**Affiliations:** ^1^Department of Neurobiology, Institute of Life Sciences, Hebrew University of Jerusalem, Jerusalem, Israel; ^2^Edmond & Lily Safra Center for Brain Sciences, Hebrew University of Jerusalem, Jerusalem, Israel

**Keywords:** cerebellum, classical conditioning, complex spike, inferior olive, Purkinje neurons

## Abstract

Recent studies demonstrate that after classical conditioning the conditioned stimulus (CS) triggers a delayed complex spike. This new finding revolutionizes our view on the role of complex spike activity. The classical view of the complex spike as an error signal has been replaced by a signal that encodes for expectation, prediction and reward. In this brief perspective, we review some of these works, focusing on the characteristic delay of the response (~80 ms), its independence on the time interval between CS and the unconditioned stimulus (US) and its relationship to movement onset. In view of these points, we suggest that the generation of complex spike activity following learning, encodes for timing of movements onset. We then provide original data recorded from Purkinje and cerebellar nuclei neurons, demonstrating that delayed complex spike activity is an intrinsic property of the cerebellar circuit. We, therefore, suggest that learning of classical conditioning involves modulation of cerebellar circuitry where timing is provided by the inferior olive and the movement kinematic is delivered by the cerebellar nuclei projection neurons.

## Introduction

Classical conditioning is a wildly studied paradigm in cerebellar research. Numerous studies have shown that the conditioned stimulus (CS) is transmitted by mossy fibers (MFs) to the cerebellar cortex whereas the unconditioned stimulus (US) is delivered by climbing fibers. Furthermore, the probability for climbing fiber response to the US is significantly reduced after learning. This learning-dependent modification gave rise to the idea that complex spike is an error signal. In recent years, new evidence show that after learning complex spikes appear after the CS and before the US (Nicholson and Freeman, 2003; Ohmae and Medina, 2015; ten Brinke et al., 2015, 2017, 2019), suggesting that the complex spike evoked by the CS provides additional information (Ohmae and Medina, 2015; Heffley et al., 2018; Popa et al., 2019; Streng et al., 2018).

**Figure 1:**
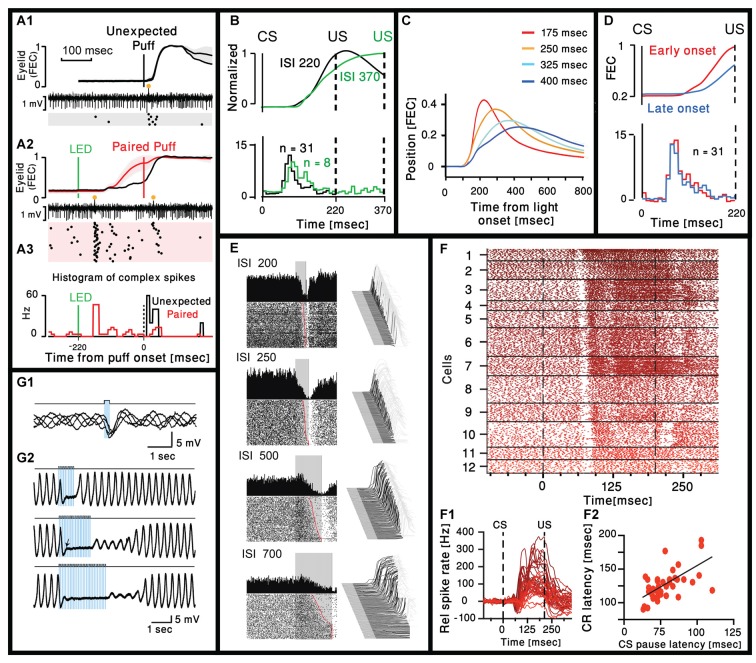
Cerebellar activity after eye-blink conditioning and related movement kinematics. **(A1)** Eyelid movements (FEC, fraction eyelid closure, presenting mean ± standard deviation, SD as shaded region) and example of simultaneously recorded Purkinje neuron (PN) activity in trials with unexpected periocular air puff. Complex spikes are marked with orange circle and their corresponding raster plot is presented below. **(A2)** Similar to **(A1)** but using paired LED and periocular air puff. **(A3)** Peristimulus time histograms (bin size = 10 ms) for the complex spikes fired in the trials corresponding to the two raster plots in **(A1)** and (**A2**; Ohmae and Medina, 2015). **(B)** Upper panel: normalized eyelid traces. Lower panel: comparison of population-averaged complex spike activity in mice trained with a 220-ms inter spike interval (ISI 220) and a mouse trained with a 370-ms ISI (ISI 370). Adapted from Ohmae and Medina (2015). **(C)** Average eyelid position, for mice trained with four different ISIs. Adapted from Chettih et al. (2011). **(D)** Upper panel: normalized eyelid traces. Lower panel: comparison of population-averaged complex spike activity in trials with early-onset and late-onset CR movements. Numbers in parentheses indicate the number of cells recorded. Adapted from Ohmae and Medina (2015). **(E)** Representative examples of eyelid (Right) and Purkinje recordings during four behavioral conditions that involved single ISIs of 200, 250, 500 and 700 ms. For each panel, a waterfall plot of all behavioral responses in the session is shown at right. For these plots, each sweep represents the response from an individual trial, first trial in front. Upward deflection represents closure of the eyelids. For each sweep the pre-conditioned stimulus (CS) portion is shown in dark gray, the time during which the CS was present is shown in black, with the post-unconditioned stimulus (US) portion of each response shown in light gray. With this arrangement, all eyelid responses during the black portions of the trace are CRs. The average response during the paired CS-US trials over the entire session is shown as a single sweep above the raster plots. For the raster plots, where the first trial is on the bottom row, each dot represents the simple-spike recording from that PN. The trials are aligned such that the CS duration is shown by the gray rectangle. These data are from four different PNs and their responses are representative of those observed for the four different ISIs. Adapted from Halverson et al. (2015). **(F1)** Combined raster plot for 12 CN neurons, ordered by the latency of their CS pause in spike activity. **(F2)** Relative spike rates corresponding to the cells shown in **(F1)**. **(F3)** CS pause latency plotted against the latency at which the CR passes 5% eyelid closure for 45 cells (ten Brinke et al., 2017). **(G1)** Superimposed voltage traces from an oscillating IO neuron. Single pulse of light elicits an IPSP in different phases of the subthreshold oscillations. **(G2)**. Three traces with trains of light stimulation given at 12.5 Hz for three different durations (0.8, 1.6 and 2.4 s). Sub-threshold oscillations were blocked for the entire duration of the train. With longer trains, the complete recovery of the sub-threshold oscillations amplitude occurs after variable delays (Lefler et al., 2014).

Ohmae and Medina (2015) and ten Brinke et al. (2019) both recorded Purkinje neuron (PN) activity in head restrained mice before and after eye-blink conditioning. PNs were considered to be eyelid-related if they reliably responded with complex spikes to the US. In both studies, after training the CS was followed by a complex spike with a delay of ~80 ms (Figures 1A1–A3). Moreover, Ohmae and Medina (2015) show that the delay to the complex spike is independent of the interval between the CS and the US (Figure 1B) and this is further supported by the work of ten Brinke et al. (2019) that used a different interval between the CS and the US. Together, these two studies show that the delay of the complex spike evoked by the CS is not related to the ***timing*** of the US.

In our view, this interesting finding sets the stage for two questions: first, of course, is what is this signal telling the brain? Is it a prediction signal that an US is about to occur? Is it a reward signal, if you behave you will avoid unpleasantness? Or is it an instruction signal: start to move now or else! Second, what and how is the circuit modified during learning to enable a complex spike response after the CS. Ohmae and Medina (2015) addressed the first question and suggested that before training the complex spike signals the novelty of a stimulus and after training it serves as a prediction error signal. ten Brinke et al. (2017) addressed the second question and suggested that the formation of MF axon collaterals is the necessary circuit modulation responsible for the generation of the CS evoked complex spike (ten Brinke et al., 2017).

In this brief perspective, we scan trough classical conditioning studies, focusing on two points. First, the initiation of movement that like the timing of the CS evoked complex spikes, is independent of the CS-US interval. Second, the kinematics of the movement is highly correlated with both the reduction in firing of PN and the increased firing of cerebellar nuclei (CN) neurons and that both are modified by the CS-US interval. We then show some results demonstrating that in anesthetized naïve animals delayed complex spikes can be elicited by stimulating either the MFs or the inferior olive (IO). Finally, we propose that the learning of classical conditioning involves modulation of the cerebellar circuitry where the climbing fibers provide timing of movement **onset** while PN *via* cerebellar projection neurons, provide the necessary information for movement kinematics.

## Cerebellar Activity During Learned Conditioned Response

### Cerebellar Activity Preceding Movement Onset

A thorough analysis of the eyelid movement during classical conditioning have been performed in many studies (Chettih et al., 2011; Halverson et al., 2015; ten Brinke et al., 2017), focusing on the time of the peak response and/or the time of maximal velocity or acceleration. Only few studies directly address the time of movement **onset**. On one hand this is rather surprising as there is a general consensus that the cerebellum is deeply involved in movement coordination, namely providing timing information. On the other hand, successful learning implies that the eyelid will close at the time of the US and therefore time of maximal closer seems more appropriate. However, we carefully examined published traces of eyelid movements and the impression is that the onset time of the movement is independent of the CS-US interval and seems to occur at a delay of ~100 ms from the CS (Heiney et al., 2014; Ohmae and Medina, 2015; Siegel et al., 2015; ten Brinke et al., 2015). This impression is supported by the work of Chettih et al. (2011), one of the few works that studied the kinematic of the response. In their study they conclude that *“… mice appear to achieve precise timing by regulating the velocity, but not the onset latency of the eyelid movement*.” This is demonstrated in Figure 1C, where traces of eyelid position in four different CS-US intervals are superimposed. Indeed, the movement onset latency (~100 ms) is independent of the CS-US interval. In a later work Ohmae and Medina (2015) differentiate early and late onset of movement (Figure 1D) where movement onset was defined by a threshold of eyelid velocity. However, careful examination of their results reveals that the time of movement onset of both early and late response is very similar. Thus, it seems likely that after learning, both the complex spike and the onset time of the eyelid movement occur after a relatively constant delay that is independent of the CS-US interval. Consequently, it is tempting to suggest that the CS evoked complex spike actually provide timing for movement initiation. This possibility is further supported by the results presented in Figure 1B, demonstrating a rear case where two different CS-US intervals where used and the movement onset time of the longer interval is somewhat delayed. Surprisingly, the time of the complex spike is also delayed and to a similar extent (Movement onset delayed by ~15 ms and peak of histogram is delayed by a single 10 ms bin).

### Cerebellar Control of Movement Kinematics

Large body of electrophysiological studies demonstrate that learning of eye blink response is associated with a reduction in simple spike activity of PN (ten Brinke et al., 2015; Jirenhed and Hesslow, 2016; Jirenhed et al., 2017; Halverson et al., 2018). Whether it is due to long term depression of parallel fibers input (Alba et al., 1994; Kim and Thompson, 1997; Koekkoek et al., 2003) or increase activity of molecular layer inhibitory interneurons (ten Brinke et al., 2015) or both, is still debated (Schonewille et al., 2011), but the relationship with movement kinematic is highly correlated. This is best demonstrated in the work of Mauk and his colleagues (Halverson et al., 2015) where the reduction in PN simple spike activity was correlated with the movement kinematics. An example shown in Figure 1E, where PN simple spike activity was measured in four different CS-US intervals. It is clear that the slower movement (longer CS-US interval) is associated with slower reduction in PN firing. However, the reduction in simple spike activity starts at the same delay from the CS (~90 ms) and again independent of the CS-US interval.

The reduction in simple spike firing should affect the firing of CN projection neurons. Indeed, ten Brinke et al. (2019) characterized the firing pattern of CN in response to CS. As shown in Figures 1F1,F2 the CN neurons respond to CS with a characteristic short pause in firing that occurs after a delay of 70–100 ms and is followed by an increase in firing rate that lasts up to the US and beyond, highly correlated with the movement parameters. They suggest that the pause elicits rebound excitation that has been shown to mediate motor activity (Witter et al., 2013). However, the pause seems identical while the firing rate is highly variable along with the movement, suggesting a significant contribution from the reduction in PN simple spike activity. The interesting observation is that delay to the pause is highly correlated with movement latency (Figure 1F3), thus strongly supporting the possibility that complex spikes triggered by the CS encode movement initiation.

### Summary and Suggestions

Summarizing this brief review demonstrates that classical conditioning is associated with the appearance of a complex spike in response to the CS. All these studies agree that these findings argue against the error signal paradigm and propose an additional or alternative role for the complex spike. We focus our review on the movement kinematic, providing evidence, whereas movement velocity is higher at shorter CS-US intervals, and movement initiation is independent of the interval. Thus, we propose that cerebellar learning of classical conditioning involve two mechanisms: learning to initiate a movement and learning the kinematics of the movement. The CS evoked complex spike signals movement onset that occurs 20 ms after the complex spike. This delay can easily be accounted for by the path from the CN neurons to the motor system that activates the eyelid muscles. The decrease in simple spike activity and the resultant increase activity of CN neurons control movement kinematics, where longer intervals are associated with slower movement, insuring eyelid closer at the right time.

## Cerebellar Circuitry Enabling CS Evoked Delayed Complex Spike Response

Understanding the circuitry that is responsible for the CS evoked complex spikes shall pave the way to decipher at least one aspect of the learning mechanisms, the timing of movement initiation. The two main inputs to the cerebellum are the MFs originating in the pontine nucleus, and the climbing fibers, originating in the IO. It is commonly accepted that the CS mainly activates MFs and that the US primarily activates the IO. Thus, the appearance of a complex spike in response to a CS represents changes within the olivo-cerebellar system.

In their work, ten Brinke et al. (2019) review different circuit pathways that may explain the circuit modifications giving rise to the CS evoked delayed complex spike. Their main suggestion is that during learning there is an outgrowth of MF collaterals to the CN (Boele et al., 2013). Thus, after learning, the MFs that are activated by the CS strongly activate specific CN projection neurons. These CN neurons activate the IO indirectly *via* the mesodiencephalic junction (MDJ). They support this hypothesis by showing that electrical stimulation in the CN can elicit EPSPs in IO neurons with a delay of 38.2 ± 14.2 ms (Bazzigaluppi et al., 2012). Although this suggestion is indeed intriguing, it requires synaptic specificity that has not been demonstrated. Moreover, if learning involves generation of new connections, how can it explain the observed fast extinction? (Medina et al., 2002). In addition, the delay from CN stimulation to the generation of complex spike is insuffcient to account for the 80 ms delay observed. Even if we add about 15 ms to account for the delay between the CS and the activation of the CN and 5 ms for the delay between the IO and PN, we are still too short.

We, therefore, suggest that the generation of the delayed complex spike reflects changes within the CN, particularly in the inhibitory projection neurons, and that the delayed complex spike is due to resetting of the olivary activity by the inhibitory input. To examine this possibility, we characterized the responses of PN and CN neurons to either MF or IO stimulation in anesthetized (Ketamine/Xylazine) mice. The MF and the IO were either electrically or optogenetically stimulated, the MF at the medial cerebellar peduncle and the IO was directly stimulated.

**Figure 2:**
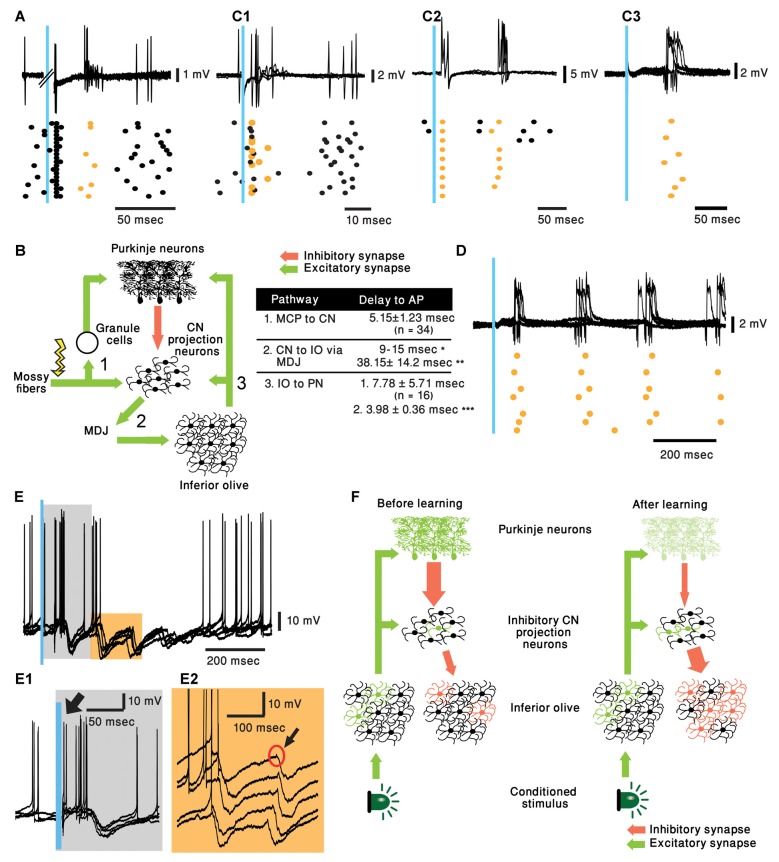
Cerebellar activity in response to mossy fiber (MF) and IO stimulation in naive mice and proposed mechanism. **(A)** five superimposed voltage responses recorded from a PN during MF stimulation (Blue bar) placed at the medial cerebellar peduncle and the corresponding raster plot. In all the panels orange stars and black circles represent complex spikes and simple spikes, respectively. **(B)** Possible pathway of MF evoked delayed complex spike response as suggested by ten Brinke et al. (2019) and a table summarizing the delays in the diagram. Delay values are collected from our recordings unless indicated otherwise. *(Ruigrok and Voogd, 1995) **(Bazzigaluppi et al., 2012) and ***(Shinoda et al., 2000). MDJ, mesodiencephalic junction. **(C1–C3)** Three different types of PN responses to IO stimulation (Blue bar), five superimposed traces and the corresponding raster plots are plotted for each type of response. Direct complex spike activation (**C1**, ~5 ms delay), direct complex spike activation and a delayed response (**C2**, ~5 and ~75 ms delay) and only delayed complex spike (**C3**, ~80 ms). **(D)** Rhythmic complex spike response to IO stimulation (Blue bar), five superimposed traces and the corresponding raster plots are plotted. **(E)** Rhythmic inhibitory bursts recorded from CN neurons in response to IO stimulation (Blue bar), five superimposed traces and the corresponding raster plots are plotted. **(E1)** Higher resolution of the gray rectangle displayed in **(E)**. Black arrow indicates direct activation of CN neuron (~5 ms). **(E2)** Higher resolution of the orange rectangle displayed in **(E)**. Black arrow indicates short excitation preceding burst of inhibitory inputs. **(F)** Suggested pathway of CS evoking delayed complex spike response after learning.

Recording from PNs reveals that as expected, stimulating the MF triggered simple spikes that appear after a delay of 4.75 ± 0.89 ms (*n* = 8). Interestingly, similar to the work of Bazzigaluppi et al. (2012), on some occasions, this response was followed by a complex spike after a delay of 37.56 ± 7.74 ms (*n* = 3, Figure 2A). This relatively prolonged delay, is likely to represent recurrent circuitry *via* the MDJ (see Figure 2B) as suggested by ten Brinke et al. (2019). However, as stated above, it can not account for the 80 ms delay of complex spikes evoked by the CS (see table in Figure 2B). Similarly, stimulating the IO resulted in direct activation of the climbing fibers evoking complex spikes after a short delay (5.15 ± 1.23 ms, Figure 2C1). Unexpectedly, in several cases an additional complex spike appeared after prolonged delay of ~75 ms (Figure 2C2, *n* = 7; mean ± standard deviation 76.22 ± 17.25 ms). This delayed response was observed also in PNs that did not directly respond to the stimulus (Figure 2C3). Furthermore, in few occasions the response was characterized by rhythmic activity at a frequency of ~5 Hz, well within the frequency of olivary subthreshold activity (Figure 2D).

To further characterize this rhythmic IO response and keeping in mind that several tens of PNs converge onto one CN neuron (Najac and Raman, 2017; Yarden-Rabinowitz and Yarom, 2017), we recorded intracellularly from CN neurons while activating the IO. Indeed, rhythmic bursts of inhibition were occasionally observed in response to IO stimulation (Figure 2E). In accordance with the delayed complex spikes in PNs, the delay to the first peak of inhibitory response was ~70 ms (Figure 2E1). It should be noted that the frequency of these events (~7 Hz) within the frequency of olivary subthreshold activity. In the presented example IO stimulation directly activated the CN neuron (Figure 2E1, black arrow). Moreover, each of the delayed inhibitory response is always preceded by small, depolarizing signal (black arrow, Figure 2E2) that represent direct olivary input to CN neurons (van der Want et al., 1989). The absence of strong inhibitory response following direct activation of the IO suggests that only a small number of olivary neurons were activated by the stimulus. On the other hand, the inhibitory delayed response suggests that it is associated with a large number of IO neurons. Thus, robust delayed olivary activity can be triggered by direct olivary stimulation and it is likely to reflect feedback activation of a larger population of neurons compared to the directly activated neurons.

## Proposed Mechanism of Classical Conditioning

In view of this brief description, it is tempting to consider the possibility that the delayed complex spike is an intrinsic property of the olivo-cerebellar network. However, the olivo-cerebellar loop is a rather temporally compact system, hence, where in the circuit can such a long delay emerge? One possible candidate is the inhibitory input from CN inhibitory projection neurons (also referred to as nucleo-olivary neurons, NO) that innervate the IO. This inhibition closely controls the functional architecture of the nucleus as was shown in an *in vitro* study (Lefler et al., 2014). This study demonstrated that the activation of the inhibitory input is sufficient to block the subthreshold activity in the IO as well as to reset the rhythm phase (Figures 1G1,G2), thereby introducing a significant delay between activation time of NO neurons and the spiking activity in the IO.

Accordingly, we propose the following sequence of events that lead to classical conditioning. In a nutshell, CS activates the IO neurons (Ju et al., 2019; Rasmussen, 2019) but the number of activated cells, as well as the reliability of the response, is rather weak. After training the same stimulus reliably activates, after a considerable delay and *via* the NO, a large population of olivary neurons. This possibility is schematically illustrated in Figure 2F. Before training (left panel) the CS (Light) activates a small number of olivary neurons (Green), that in addition to activating the PNs, also innervate the NO projection neurons (De Zeeuw et al., 1997). However, under naïve conditions, the NO neurons are inhibited by the PN preventing them from delivering a significant output to the IO. After learning (right panel), there is a reduction in PN activity, commonly accepted paradigm of cerebellar learning. This reduction relieves the NO neurons from inhibition, consequently, the IO input to the NO neurons becomes more efficient and more reliably activates the NO neurons. Again, the involvement of the inhibitory feedback of the NO in cerebellar learning processes has been well established (Andersson and Hesslow, 1987; Andersson et al., 1988; Llinás and Welsh, 1993). As a result, the feedback inhibition to the IO resets the olivary activity and thus, synchronously activates a large population of IO neurons (red cells) at a delay of 70–80 ms.

To conclude, recent classical conditioning studies presented the emergence of a delayed complex spike response following a CS. Reviewing evidence in the literature implies that this delayed complex spike response signals the initiation of movement whereas the kinematics of the movement that is acquired during learning is determined by Purkinje and CN neuronal activity. Our presented data argue that the delayed complex spike is a result of modifications in the activity of CN inhibitory projection neurons and not a result of a feedforward excitation *via* MFs.

## Data Availability Statement

All datasets generated for this study are included in the manuscript.

## Ethics Statement

The animal study was reviewed and approved by Hebrew University of Jerusalem Animal Care Committee.

## Author Contributions

YY-R recorded from PN and CN neurons and analyzed the data. YY-R and YY conceived the idea and wrote the manuscript.

## Conflict of Interest

The authors declare that the research was conducted in the absence of any commercial or financial relationships that could be construed as a potential conflict of interest.
